# Three-Dimensional Structure of the Enveloped Bacteriophage Φ12: An Incomplete T = 13 Lattice Is Superposed on an Enclosed T = 1 Shell

**DOI:** 10.1371/journal.pone.0006850

**Published:** 2009-09-03

**Authors:** Hui Wei, R. Holland Cheng, John Berriman, William J. Rice, David L. Stokes, A. Katz, David Gene Morgan, Paul Gottlieb

**Affiliations:** 1 Department of Microbiology and Immunology, Sophie Davis School of Biomedical Education, The City College of New York (CCNY), New York, New York, United States of America; 2 Nanoscience Center, Department of Chemistry, Indiana University, Bloomington, Indiana, United States of America; 3 Department of Cellular and Molecular Biology, University of California at Davis, Davis, California, United States of America; 4 Structural Biology Program, Skirball Institute of Biomolecular Medicine, New York University School of Medicine, New York, New York, United States of America; 5 The New York Structural Biology Center, New York, New York, United States of America; 6 Institute for Ultrafast Spectroscopy and Lasers, The City College of New York, New York, New York, United States of America; University of Minnesota, United States of America

## Abstract

**Background:**

Bacteriophage φ12 is a member of the *Cystoviridae*, a unique group of lipid containing membrane enveloped bacteriophages that infect the bacterial plant pathogen *Pseudomonas syringae pv. phaseolicola*. The genomes of the virus species contain three double-stranded (dsRNA) segments, and the virus capsid itself is organized in multiple protein shells. The segmented dsRNA genome, the multi-layered arrangement of the capsid and the overall viral replication scheme make the *Cystoviridae* similar to the *Reoviridae*.

**Methodology/Principal Findings:**

We present structural studies of cystovirus φ12 obtained using cryo-electron microscopy and image processing techniques. We have collected images of isolated φ12 virions and generated reconstructions of both the entire particles and the polymerase complex (PC). We find that in the nucleocapsid (NC), the φ12 P8 protein is organized on an incomplete T = 13 icosahedral lattice where the symmetry axes of the T = 13 layer and the enclosed T = 1 layer of the PC superpose. This is the same general protein-component organization found in φ6 NC's but the detailed structure of the entire φ12 P8 layer is distinct from that found in the best classified cystovirus species φ6. In the reconstruction of the NC, the P8 layer includes protein density surrounding the hexamers of P4 that sit at the 5-fold vertices of the icosahedral lattice. We believe these novel features correspond to dimers of protein P7.

**Conclusions/Significance:**

In conclusion, we have determined that the φ12 NC surface is composed of an incomplete T = 13 P8 layer forming a net-like configuration. The significance of this finding in regard to cystovirus assembly is that vacancies in the lattice could have the potential to accommodate additional viral proteins that are required for RNA packaging and synthesis.

## Introduction

The cystoviruses (φ6–φ14) are a unique group of viruses that infect strains of the plant pathogen *Pseudomonas syringae* pv *phaseolicola*. They have been very useful research models for elucidating the replication mechanisms of RNA viruses [1]. In particular, the overall replicative mechanism and the multishell structure of cystoviruses are analogous to members of the *Reoviridae* family [1]. Both virus families package their mRNA as precursors to the double-stranded RNA (dsRNA) genomic segments. Within the cystovirus family, all species share a very similar genetic organization and encode a comparable set of proteins [2]. In addition, the RNA-directed RNA polymerase (RdRP) is structurally and mechanistically related to the comparable enzyme of the flaviviruses and has been used to study *de novo* initiation of viral RNA synthesis [3]–[5].

**Figure 1:**
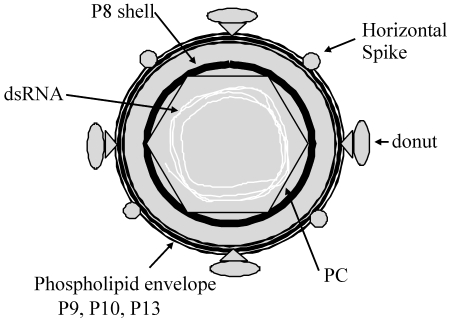
Schematic representation of the φ12 virus particle. The arrows and labels designate the major structural components of the virus particle. The nucleocapsid is composed of the P8 layer surrounding the two proteins that constitute the polymerase complex (PC). The dsRNA segments are shown as light shaded coiled lines packaged within the PC.

A schematic of the structure common to all of the cystovirus species is depicted in Fig. 1 and summarized by Gottlieb [6], [7]. Three segments of dsRNA are packaged, replicated, and transcribed within the inner viral shell, the polymerase complex (PC) [8]. The three dsRNA segments are shown in a circular format in the schematic diagram. The PC, as defined in φ6, is assembled from four viral proteins (P1, P2, P4, and P7) that are arranged in a dodecahedral conformation [9]. In the work presented here, we find that the φ12 PC is composed of proteins P1 and P2, represented by the hexagon in the schematic. The P1 protein is the main structural element of the PC and is responsible for the organization of the dodecahedral assembly in φ6, existing as two structurally non-equivalent monomers, named A and B [10]. P2 is an RNA-dependent RNA polymerase [11]. The dsRNA-packaged PC is subsequently covered by a P8 protein shell, which together with P4, P5, and P7, constitute the viral nucleocapsid (NC). The P8 proteins of the NC shell are arranged as trimers organized in a T = 13 icosahedral lattice [10], [12]. The P8 shell is shown as the dark circle in the schematic. P4 is a hexameric complex that possesses NTPase activity and is located at the 5-fold symmetry axes of the NC [13], [14]. P7 is a dimeric protein and maintains RNA packaging efficiency [15]. All cystoviruses are enveloped by a phospholipid envelope membrane which constitutes the outer virus layer and contains viral encoded proteins P3, P6, P9, P10, and P13 [9], [16], [17]. Protein P12 is a non-structural protein that mediates the membrane envelope acquisition that surrounds the viral NC. It is also responsible for inserting the viral proteins into the phospholipid envelope [17], [18]. A protein complex composed of membrane proteins P3 and P6 constitute the viral attachment apparatus [17], [19], [20].

Cryo-electron microscopy (cryoEM) has previously been used to reveal the organization of proteins making up the isolated NC of φ6 as well as the docking of the hexameric ATPase at the five-fold vertex of the NC [21]. These studies relied on the icosahedral symmetry innate to the NC, either for averaging of symmetry-related subunits or for orientation of the PC at the 5-fold vertex. Recently we have utilized cryo-electron tomography to study the shape and distribution of the non-symmetric components of the intact φ12 cystovirus [22]. In our 3-D reconstructions, the φ12 virus is seen to have two discreet shells resulting from the NC and the membranous envelope, respectively. In prior studies, our tomograms revealed periodic connections between the NC and the inner surface of the envelope that appear to maintain the NC's centralized position [22]. The envelope's outer surface is decorated with two types of protruding densities: elongated structures that are closely associated with the membrane surface; and distinctive “donuts” that are set further away from the surface (Fig. 1).

In this study, we examine the entire φ12 virus particle and present single particle reconstructions that describe the symmetrical viral components. We show that the P8 protein layer is organized on an incomplete T = 13 icosahedral lattice in which the symmetry axes of the T = 13 layer and the enclosed T = 1 layers are superposed. Our observations expand on previous architectural descriptions for the NC of species φ6 and φ8. The organization of the NC into layers of P8 and P1 is similar to what is seen in φ6 but our work will show that the details of the P8 layer in φ12 are quite distinct from what is found in φ6. Previous single particle analysis revealed that the φ6 NC consists of P8 trimers organized on a T = 13/ icosahedral lattice interrupted at the 5-fold vertices by P4 hexamers [10]. However an equivalent P8 T = 13 layer was absent in species φ8 [23]. In regard to the NC organization, our results suggest that φ12 has a structural state that is intermediate between that of φ6 and φ8. We find that in φ12, the P8 icosahedral-lattice is an open net-like structure and we hypothesize that the vacancies found in the incomplete P8 T = 13 layer could provide available sites for the insertion of two additional viral proteins.

## Results and Discussion

### Central sections of the φ12 PC indicate that it is composed of two proteins

**Figure 2:**
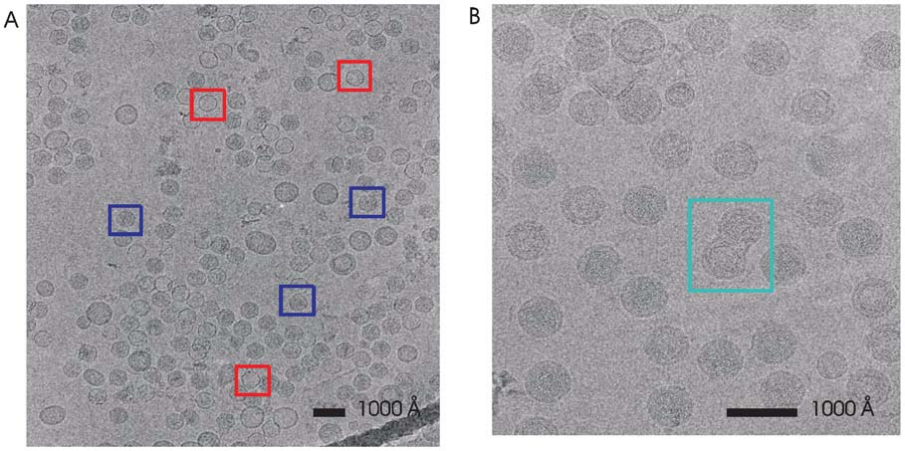
Representative images of virus samples containing empty and full PC (panel A) and intact virus (panel B). The PC sample contains empty PC particles (examples are shown in red boxes), full PC particles (examples are shown in blue boxes) and a variety of vesicles that are likely to contain protein and/or lipid. The intact virus particles are larger and clearly surrounded by a bilayer structure. Double virus particles (turquoise box) occur at the rate of about one double particle per micrograph. The scale bar in both images represents 1000 Å.

Our virus preparations contained polymorphic forms consisting of either intact virus or particles stripped of the envelope. We expected that these particles would be similar to the NC particles made from φ6; however, we were surprised that our core structures, either filled or empty of dsRNA, were composed of only two proteins, P1 and most likely P2. Therefore, we note that PC particles from φ12 are mostly likely composed of only 2 proteins, P1 and P2, while the PC from φ6 is composed of 4 proteins, P1, P2, P4 and P7. Indeed the processed images contain no complete NC when acquired from viral samples with the envelope removed, suggesting that the P8 layer has an intimate envelope association. Therefore, we were able to select significant numbers of PC images that either lacked or contained dsRNA. While we were unable to determine if the polymorphism was the consequence of incomplete assembly or viral disassembly, we exploited this fortuitous result by analyzing all forms to produce the reconstructions. Fig. 2A shows representative images from the sample containing both empty and full PC. The intact viruses are surrounded by a membrane envelope and are larger than the NC (Fig. 2B). Particles containing multiple NCs occurred at the rate of about one per micrograph. The lipid bilayer appears to form a continuous layer around pairs of NCs indicating that they are not overlapping particles [22].

**Figure 3:**
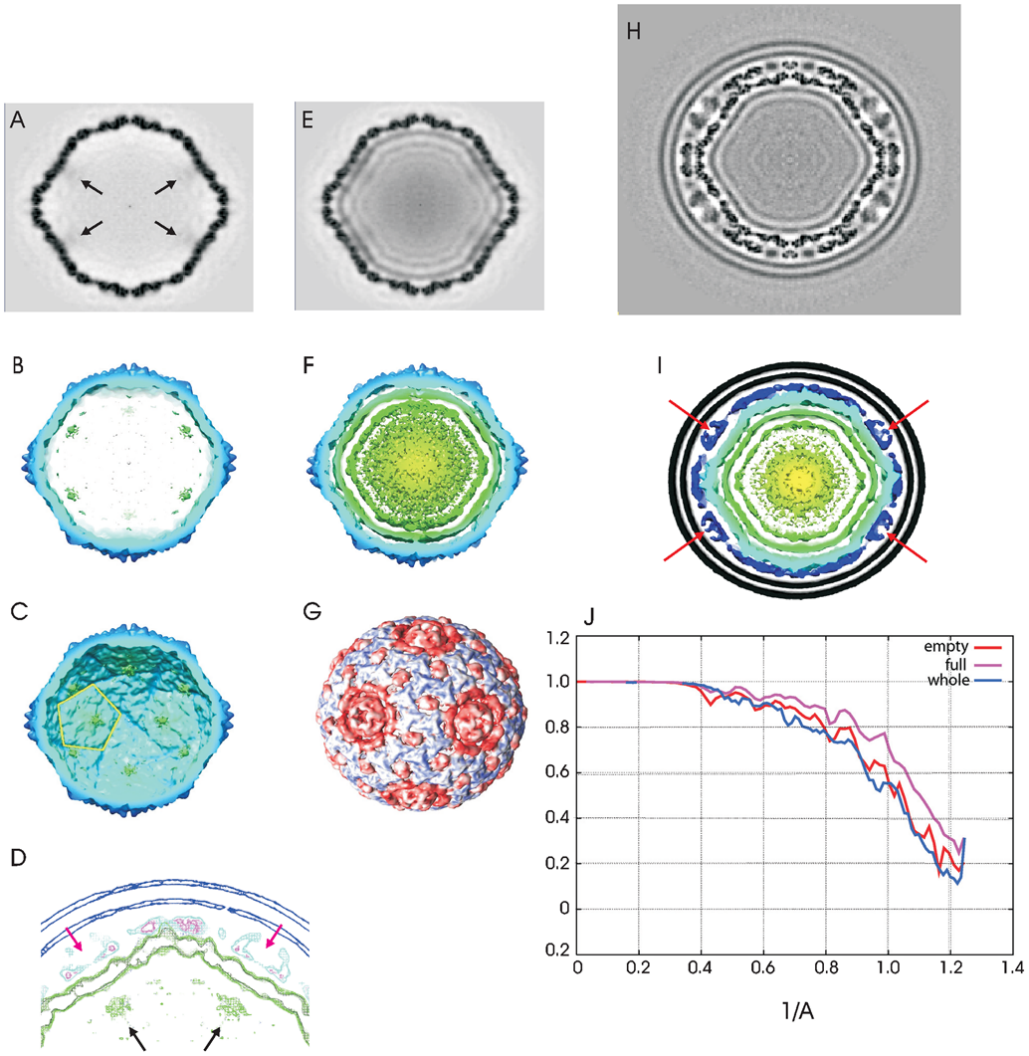
Central sections and surface representations of empty PC, RNA filled PC, and the entire virus particle made after enforcing 532 point group symmetry (icosahedral and dodecahedral symmetry). A. Empty PC reconstruction. The RdRP, P2, densities are indicated with arrows. B. Inner surface of the empty PC. Positions of the RdRP, P2, are indicated in green. C. Cross-section of the empty PC showing the P2's located at the 5 fold axis of symmetry (yellow pentagon). D. Mesh-surface representation of the cross-section projection rendering of two 5-fold axes from the entire virus particle showing the P2 RdRP directly below the P4 turret. E. dsRNA-filled PC reconstruction. F. Cross-section of the filled PC. The RNA segments are shown in green. G. The surface of the NC was generated from the reconstruction of the intact virus particle after removal of the lipid bilayer. The blue features represent the steepest density gradient (i.e. very well defined densities) while the red features represent the shallowest density gradient (i.e. less well defined features). H. Intact virus particle reconstructions I. Cross-section of the intact virus particle. The P4 NTPases are shown by red arrows. The other dark blue layer represents P8. Black is the envelope, dark blue is the P8/P4 layer, light blue is P1 layer, green is RNA. J. The result of 3 separate FSC determinations. The resolution of both the empty and full PC and intact virus is better than 10 Å in both these data sets. The x-axis is labeled in 1/Å.

As compared to the empty PC (Figs. 3A,B,C) both the full PC and intact virus particles (Figs. 3E, F and 3H, I) show the internal layering of the dsRNA genome which provides a measure of the RNA packaging density. Such layering is commonly seen in icosahedral virus reconstructions. The average spacing between the layers is approximately 32 Å and is compatible to what is seen in φ6 [10], φ8 [23], and rotavirus reconstructions [24]. The condensed RNA remained concentric with respect to the particle center suggesting that the positively charged P1 holds the RNA in a rigid position with respect to the capsid's inner surface [25]. This is similar to the association of dsRNA segments with the rotavirus VP1/VP3 PC [26], [27].

The empty PC particle shows the presence of a protein directly located below the 5-fold symmetry axes. Central sections of reconstructions constrained to 532-point group symmetry (icosahedral and dodecahedral) are shown in Fig. 3. The reconstruction of the empty PC shows (see Fig. 3A) densities directly beneath the central position of the 5-fold symmetry axes. The particles are in one of the standard icosahedral virus particle orientations with the 2-fold symmetry axes aligned along the x-, y- and z-axes. These sections come from reconstructions that have been contrast transfer function (CTF) corrected. By inference, we identify this protein as the RdRP, P2. There should be 12 copies of P2 in the PC beneath each P4 hexamer at the 5-fold axes. Although its density is smeared out by misapplication of 5-fold symmetry to the single copy of P1 near the 5-fold icosahedral axis, the density along the 5-fold symmetry axes and inside the layer of P1 protein clearly indicates the presence of an additional protein which we tentatively identify as P2 protein (Figs. 3A–C). Sen et al. reported that in the recombinant φ6 PC P2 densities were observed at the 3-fold axis in particles lacking dsRNA [28]. They proposed that at viral maturation the P2 molecules rotate to occupy positions close to the 5-fold vertices in order to perform the RNA replication and transcription functions. Our φ12 images are derived from assembled viral particles that have undergone the maturation process hence the data showing P2 near the five fold axis, supports the view that P2 is mobile within the P1 shell. It also appears that the five fold vertices are likely to be occupied by P2 in the mature φ12 virus in contrast to recombinant φ6 PC that does not assemble with complete efficiency [28]. In addition, φ6 temporal control of transcription is regulated by a host-cell protein, YajQ, that conceivably could be assembled in proximity to the RdRP in the PC [29]. Our previous work has demonstrated by SDS-PAGE analysis of gradient isolated φ12 viral particles that their NC showed only the viral components while no host-derived proteins were evident [7], [30]. The φ12 P2 protein is seen to be located directly beneath the P4 turret (see Fig. 3D) placing it central to the vertex portal (site of viral RNA entry). This is in contrast to the reovirus RdRP λ3 that lies to one side of the five-fold axis and therefore off-center in relation to the λ2 turret position [31].

### The P8 layer forms an incomplete icosahedral lattice

**Figure 4:**
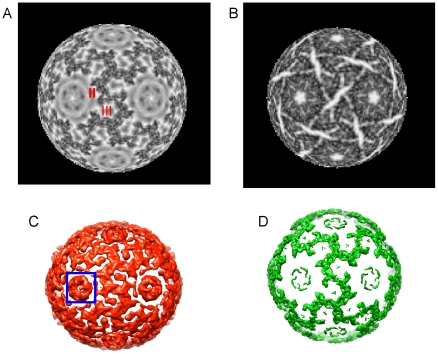
Details of the φ12 NC structure. A. Density layer at a constant radius that cuts through the NC (P8 protein) layer. The radius of the density layer is 264 Å through the NC P8 layer. B. Density layer at a constant radius (224 Å) that cuts through the PC (P1 protein layer). These figures (A and B) were generated using robEM. P8 layer and P4 proteins viewed along one of the icosahedral 2-fold axes. Panel C shows a rendering of the P8/P4 layer at a low but informative threshold (red contour) and a very high threshold (D) (green contour) that shows the most-dense features. The 5-fold turret-like features (left one is indicated with a blue box in panel C) correspond to hexamers of P4.

Fig. 4A shows a radial surface cutting through the layer of P8 protein. The P8 trimers are organized using a T = 13 surface lattice. The fact that P8 trimers do not cover the entire surface at this radius shows that the layer is “incomplete.” In φ6 the layer of P8 trimers is organized as a T = 13 icosahedral lattice interrupted only at the 5 fold vertices by the P4 hexamer [10], [23]. The copy number of the P8 proteins is 600 in φ6 [7], [9], [32]. Due to the structural symmetry between φ6 and φ12, we estimate the φ12 has a comparable number of P8 trimers. The density that we identify as the φ12 P8 is arranged in a similar manner to the φ6 P8 density [23]. In addition the placement of the P8 trimers in the T = 13 lattice of φ6 and φ12 is similar. The φ8 P8 layer is essentially absent and 60 copies of a minor 11 kDa protein are loosely bound to the PC shell [23]. Therefore our data demonstrates that although φ6, φ8, and φ12 all have an incomplete T = 13 layer of P8 trimers at this position in their NC [10], [23] the details of the protein layer in the three viruses are very different. Fig. 4B shows a radial surface cutting through the PC (P1) protein layer. This is a similar view to that of virus φ6 where this layer has been segmented into non-symmetric dimers of protein P1 [10]. The two P1 protein layers are very similar in overall organization.

Figs. 4C, D show the P8 layer viewed along one of the icosahedral 2-fold axes in an isosurface rendering performed using UCSF Chimera software (www.cgl.ucsf.edu/chimera). Panel C of Fig. 4 shows a rendering of the P8/P4 layer using a low threshold. The turret-like features appear most strongly in Fig. 4C (highlighted by a blue box in Fig. 4C) and correspond to hexamers of the NTPase P4. A comparison of Figs. 4C to 4D shows that the type II and type III holes of the T = 13 lattice are occupied by novel densities. The borders of the type II and III holes are defined by the S, Q, R, and T P8 trimers, as defined by Butcher [10], [12]. Huiskonen et al [10] shows that the type II and type III holes are unoccupied in φ6. The enforced 5-fold symmetry shows these hexameric NTPase features as strongly 5-fold. The 5-fold symmetry of the NC is mismatched to the P4 hexamer as shown by de Hass [21]. X-ray crystallography of the φ12 shows P4 is a hexameric protein [14]. In contrast to the low threshold rendering, panel 4D shows the same view contoured to show only the most-dense features of the layer. The denser features form an open net-like conformation (Fig. 4D). The representation very clearly shows that the P8 protein trimers are arranged on an incomplete T = 13 icosahedral lattice (open net-like structure in Fig. 4D) and are the most dense and best defined structures in this layer of the virus. The 5-fold symmetry axes in the P8 layer and the enclosed T = 1 layer (composed of P1 protein) superpose.

### The relative positions of the 5-fold vertex elements; docking atomic models

**Figure 5:**
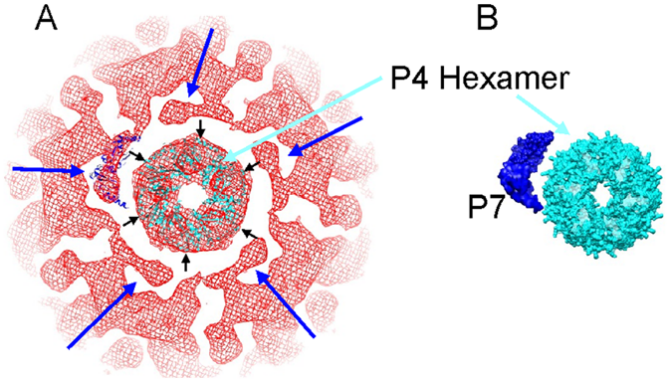
Space filling model of the NC vertex structure. A. At the five fold axis the P4 hexamer is seen to fit well in the EM-based reconstruction (inner red ring in the Figure). The atomic model of the packaging factor protein, P7, is shown in dark blue ribbon rendering beside the hexameric structure of the P4 NTPase (light blue) and has been placed in approximately the same position as the densities surrounding the P4 hexamer. The rigid crystal model of the dimeric P7 protein appears to surround the densities in immediate proximity to the hexameric P4. The less structured region along with the imposed five fold symmetry accounts for the imprecise fit. B. The isolated hexameric P4 and dimeric P7 atomic models are shown in their relative positions in the packaging vertex.

The structural relationships of the protein components at the 5-fold symmetry axes were determined. Densities representing the hexameric P4 NTPase were noted beneath the lipid bilayer and at the same radius of the P8 protein trimers (Figs. 3H, I). These structures sit along the 5-fold symmetry axes of both the NC and PC protein layers and appear pentameric in these image reconstructions where 5-fold symmetry has been imposed (Figs. 3G and 4C). The P4 has been shown to be hexameric and the corresponding symmetry mismatch relative to the neighboring structural elements in the capsid are essential to its function as part of the RNA PC [21]. We were able to manually dock the x-ray diffraction structure, PDB entry 1w4c, [14] of the hexameric NTPase into Fig. 5A (turquoise region near the center of image). The 5-fold vertices of the icosahedrally symmetrical nucleocapsid are indicated by blue arrows in Fig. 5A and the 6 NTPase subunits as black arrows. The hexamer is seen to fit at the vertices and highlights the symmetry mismatch between the 6 P4 subunits and the surrounding NC elements with the enforced 5-fold symmetry. Notably, the P4 hexamer is surrounded by five densities lying between the P4 NTPase at the NC vertex and the nearest trimers of P8 protein. These densities are novel in the sense that nothing comparable has been reported from the structures of φ6 or φ8. They are seen to occupy the position of the missing “P8 P trimer” in the incomplete T = 13 lattice of the P8 protein layer from φ6 [33]. This is also the position referred to as “type II holes” in the description of the P8 layer of φ6 described by Huiskonen et al. [10], [23].

We placed an atomic model of the dimeric P7 structure [PDB entry 2q82, [15]] in the cryo-EM density. We also placed an the X-ray structure of the hexameric NTPase [PDB entry**?**] in the approximate position of the cryo-EM densities. The P7 dimer doesn't precisely fit the density from the EM reconstruction (Figs. 5A, B) but it does appear that the P7 structure from X-ray diffraction (shown in dark blue in Figs. 5A, B) could reasonably sit in the location between the P4 structure and the P8 layer; especially given the effect on occupancy and disorder that the significant symmetry mismatch must entail. Huiskonen [10] has shown that the density assigned to P7 can not reasonably be assigned to P8. We attribute the apparent size difference to the disorder, a result of the symmetry mismatch and inherent flexibility within P7 [15]. Interestingly, the P7 dimer has been found to poorly associate with recombinant φ12 particles that lack P8 (Gottlieb, unpublished). In φ6, P7 is found to be accessible to anti-P7 antibodies on the NC surface and P8 is released [34]. These observations imply that in φ12, the P8 shell is required for the integrity of the entire PC; and P7 could occupy the position provided by the type II hole in an incomplete T = 13 lattice. Therefore, these holes are occupied by additional proteins in φ12 but empty in φ6.

### A second minor protein is noted in the intact virus

**Figure 6:**
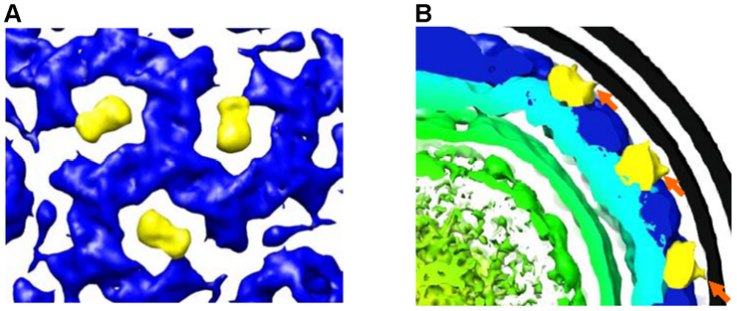
The densities tentatively assigned to P5 (yellow). The colors designating the RNA, P8 layer, and RNA correspond to those used in Figure 3, panel H. A. A top view along the three fold axis reveals novel densities surrounding the symmetry axis. The contour levels are identical to those shown in Figure 4A and indicate that the novel densities that surround the 3-fold axis are significantly less dense than the P8 proteins in the NC layer. B. The membrane association of these densities has been highlighted by using a relatively low threshold (only applied to the P8 protein layer) for both the lipid bilayer and this outer layer of the NC. This low threshold reveals the connections between the bilayer and the novel densities we tentatively assign to P5 (orange arrows). We believe that it is this membrane association that causes P5 to be removed by Triton X-100 [30].

In addition to the features that are attributed to P7 and surround the 5-fold P4 turrets and “fill in” the gap in the incomplete T = 13 P8 layer, we note a second set of novel densities that surround the 3-fold axis at a slightly larger radius than the P8 layer (Fig. 6A). When these weak densities are examined with regard to the lipid bilayer we see that these are the only features in the intact virus structure that extend towards the lipids and that they are less tightly associated with the NC or PC structures (Fig. 6B). These properties suggest that the protein might be the murein peptidase P5. Furthermore, the P5 protein is removed from the viral nucleocapsid after Triton x-100 treatment [7], suggesting some association with the lipid bilayer. This is a surprising result in that it strongly suggests that P5 could be symmetrically placed in the viral particle. Our image reconstruction allows a determination of 60 P5 protein units per particle. This is less than the original estimate of 89 by isotope labeling in φ6 [32]. These densities, that we potentially assign as P5, appear to sit with the equivalent of the φ6 NC type III holes as described by Huiskonen et al. [10]. This is of interest in that in the φ6 virus the type III holes are seen to be empty. On an evolutional level the holes could provide sites able to accommodate proteins in conformations specific to the replication mechanism for particular cystovirus species. Although our results show 60 symmetrically located P5 copies, the volume enclosed indicates partial P5 occupancy and disorder. Therefore we acknowledge that is possible that all the P5 copies do not exclusively occupy the 3 fold location accounting for the earlier estimate of 89 copies per virus particle [32].

## Materials and Methods

### Bacterial strains

*P. syringae* pv. *Phaseolicola* HB10Y (HB) is the host of phage φ6 and was utilized as a phenotypic screen based on its noninfectivity by φ12. *P. syringae* pv. *Phaseolicola* strain LM2333 is a mutant of HB which is productively infected by φ12 [2].

### Media and Chemicals

The media used to grow the HB host strain was Luria-Bertani (LB) supplemented with 50 µg/ml ampicillin. Buffers ACN (10 mM KPO_4_ (pH 7.5), 1 mM MgSO_4_, 200 mM NaCl, and 0.5 mM CaCl_2_) and P (20 mM Tris-HCL (pH 7.5) 150 mM NaCl, 0.5 mM CaCl_2_, and 1 mM MgCl_2_) were used for the suspension of purified bacteriophage. n-Octyl-beta-D-glucopyranoside (OG) and Octylphenolpoly (ethylene glycol ether) (Triton X-100) was purchased from Sigma (MP Biomedicals, Eschwege, Germany).

### Preparation of pure virus

φ12 were plated into soft agar with a culture of LM2333 that had been grown overnight. A total of 20–40 plate lysates were incubated overnight at room temperature. The top layer of agar, which contained the φ12 bacteriophage, was then collected and the cell debris and agar were removed by centrifugation in a Sorvall SS-34 rotor at 15,000 rpm, 15 min at 4°C. Phage was collected by centrifugation of the supernatant in a Beckman TI270 rotor at 33,000 rpm, 1 hr at 4°C. The resulting pellet was resuspended in 1 ml of buffer P. The bacteriophage sample was next layered on a 10–30% sucrose gradient and centrifuged at 23,000 rpm for 1 hr at 23°C using a Beckman SW 50.1 rotor. The band of bacteriophage was detected by light scattering and was collected by needle puncture. After pelleting and resuspension in buffer P, final purification of the bacteriophage was accomplished by equilibrium centrifugation in a 40–60% sucrose gradient using a Beckman SW 50.1 rotor at 23,000 rpm, overnight at 4°C. The purified bacteriophage band was located as above and was finally centrifuged in a Beckman TI270 rotor at 33,000 rpm, 2 hr at 4°C and resuspended in 100 µl buffer P.

### Cryo-Electron Microscopy

A suspension of bacteriophage φ12 was placed onto glow-discharged, perforated Quantifoil grids, blotted and rapidly plunge-frozen in liquid ethane cooled with liquid nitrogen. Images were recorded on Kodak SO-163 film (Kodak, Rochester, NY) using standard minimal dose techniques at a nominal magnification of 50 kx at the film plane using a Tecnai F20 electron microscope (FEI, Inc, Hillsboro, OR) operating at 200 kV. A defocus range of 0.6 to 3.0 µm was used to record over 200 micrographs, which were digitized and evaluated before image processing.

### Image processing

Electron micrographs were digitized using a Heidelberg D-8200 drum scanner (Heidelberg USA, Inc., Kennesaw, GA) with a step size of 10 µm. Images were binned 2×2 for a final (un-calibrated) pixel size of 4.0 Å/pixel. Images of well-separated single virus particles (Fig. 2) were manually selected from the digitized micrographs using the robEM software (http://cryoem.ucsd.edu/programs.shtm). The particles selected from a single micrograph were further processed using robEM to determine each micrograph's overall quality in regard to astigmatism, drift, and defocus. Over 8000 157 by 157 pixel images of empty or dsRNA-filled φ12 PC particles were selected from about 70 micrographs and over 4800 221 by 221 pixel images of the intact virus were selected from about 40 micrographs. The defocus determined directly from these images ranged from ∼0.2 to 4.0 µm underfocus.

### Reconstructions of φ12 virus particle

We first generated initial models of the empty φ12 PC structure (Fig. 2A) that obeyed icosahedral/dodecahedral symmetry (*i.e.*, 532 point group symmetry) using both the combined self- and cross-common lines procedures obtained with the Polar Fourier Transform (PFT) programs, obtained by following Crowther [35] and the *starticos* procedure found in the EMAN1 software package [36]. Both of these approaches generate their initial models using a very small sub-set of the available particles (Table 1). These two initial models differed significantly from each other. However, after aligning the full set of empty φ12 PC particles (∼2600 particles) to either initial model (using one cycle of the PFT programs or multiple cycles of EMAN1's *refine* command), essentially identical reconstructions were obtained. The EMAN1 programs were also utilized to generate a completely independent initial model from a small number of the dsRNA-filled φ12 PC particles (Fig. 2A) and this model was aligned to the full set of filled particles (Table 1). Except for the density inside the reconstruction that can be attributed to the dsRNA segments, this reconstruction appeared identical to that of the empty PC particles (Fig. 3 A,D). The volumes were not masked when determining the FSC curves. The full sets of particles were split into halves using an option in the reconstruction software contained in the PFT package for generating such half data sets. Actual FSC calculations were done using multiple programs designed to do FSC comparisons of two volumes. The CTF correction was done using standard methods contained in the PFT package and implemented during the reconstruction step.

**Table 1:** Reconstruction Statistics.

	Full PC	Empty PC	Whole Virus
Number of particles	5394	2674	4819
Number of micrographs (same images for Full and Empty)	74	74	38
Pixel size (Å/pixel) (same for Full and Empty PC)	4	4	4
Defocus (um) (same for Full and Empty PC)	1.7–4.0	1.7–4.0	0.6 to 3.5
Number of particles used in initial models:			
PFT programs	<20	not done	not done
EMAN	150	150	Not done
Resolution (Å)	9.0	9.4	9.5

In order to process the images of intact φ12 virus particles, we next followed the steps outlined above for the φ12 PC particles. However we were unable to produce a reasonable reconstruction using either of those methods or similar methods after classification of the intact virus particles using multi-variate statistical approaches [37], [38]. We were ultimately able to produce a reconstruction that was further refined by using the reconstruction of the dsRNA-filled φ12 PC particle as the initial model for a single cycle (see below) of alignment using the PFT programs. To accomplish this, the PC reconstruction was floated into a volume commensurate with the larger size of the intact φ12 virus particles, and the outermost radial limit was set to include the entire PC particle but none of the additional material found at higher radius in the intact φ12 virus. In addition, we limited the highest resolution during this initial alignment step to 30 Å, a value which should maintain the overall molecular envelope of the PC reconstruction without allowing too much of its detail to affect the alignment process.

After alignment of the full set of intact φ12 virus particles to this model and the generation of an initial reconstruction based on that alignment, it was clear (data not shown) that there were significant features present in the reconstruction beyond the radial limit of the φ12 PC particle used during particle alignment (most significantly the membrane bilayer and material directly beneath it). Therefore a second alignment cycle was run using an outer radial limit that included the lipid bilayer and limited the resolution to 25 Å. This alignment cycle produced clear indications that the structure of the intact φ12 virus would extend to at least 15 Å according to the FSC criterion.

In order to assure ourselves that this approach was not producing model bias, we repeated the process using both an appropriately scaled reconstruction of BK virus [39], a human polyoma virus, and a failed reconstruction produced while evaluating different point group symmetries for the φ12 PC particles. Both these models lead to reconstructions that showed the viral lipid bilayer, but neither reconstruction had any other significant features beyond the φ12 PC layer (Fig. 3H, I). In addition they did not have resolution beyond the limit set during alignment. Subsequent cycles of alignment behaved as if an incorrect model were being forced onto the intact φ12 virus images (*i.e.*, discrete structures never appeared, resolution was exactly set by the limits used during alignment, *etc.*). These observations convince us that the bootstrapping procedure starting from the φ12 PC structure had produced the correct model. Fourier shell correlation (FSC) analyses of these three reconstructions indicate that the resolution of all three is similar (Fig. 3J) and using the FSC criterion of 0.5 we calculated that the resolution of the empty PC is ∼9.4 Å, that of the full PC is ∼9.0 Å and that of the intact particle is ∼9.8 Å.
